# Patient experiences of buprenorphine dispensing from a mobile medical unit

**DOI:** 10.1186/s13722-024-00484-4

**Published:** 2024-07-18

**Authors:** Sarah E. Messmer, Abigail T. Elmes, Alexander F. Infante, Anna Patterson, Mackenzie Smith, Albert Leon Murphy, Antonio D. Jimenez, Stockton Mayer, Dennis P. Watson, Kevin Whitfield, Steven J. Fisher, Jennie B. Jarrett

**Affiliations:** 1 Department of Medicine, University of Illinois at Chicago, Westside Research Office Building, Rm 256, 1747 W Roosevelt Rd, Chicago, IL 60608 USA; 2 Department of Pharmacy Practice, College of Pharmacy, University of Illinois at Chicago, 833 S. Wood St, Chicago, IL 60612 USA; 3 College of Medicine, University of Illinois at Chicago, 1853 W Polk St, Chicago, IL 60612 USA; 4 School of Public Health, Community Outreach Intervention Projects, University of Illinois at Chicago, 1603 W Taylor St, Rm 851, Chicago, IL 60612 USA; 5 Department of Medicine, University of Illinois at Chicago, 808 S Wood St, Rm 888, MC 735, Chicago, IL 60612 USA; 6 Center for Dissemination and Implementation Science, Chestnut Health Systems & University of Illinois at Chicago, 221 W Walton St, Chicago, IL 60610 USA; 7 College of Pharmacy, Department of Pharmacy Practice & American Medical Association, University of Illinois at Chicago, 833 S. Wood St, Chicago, IL 60612 USA

**Keywords:** Opioid use disorder, Buprenorphine, Mobile medical unit, Low-threshold, Harm reduction

## Abstract

**Background:**

Overdose deaths continue to rise within the United States, despite effective treatments such as buprenorphine and methadone for opioid use disorder (OUD). Mobile medical units with the ability to dispense buprenorphine have been developed to engage patients and eliminate barriers to accessing OUD treatment. This study reports survey responses of patients of a mobile medical unit dispensing buprenorphine in areas of Chicago, IL with high overdose rates.

**Methods:**

All patients who were dispensed buprenorphine via the mobile medical unit were invited to participate in a 7-item anonymous survey between May 24, 2023, and August 25, 2023. The survey included 5-point satisfaction scale, multiple-choice, and open-ended questions. Outcomes included satisfaction with buprenorphine dispensing from the mobile medical unit, satisfaction with filling buprenorphine at a pharmacy in the past, barriers experienced at pharmacies when filling buprenorphine, and whether the client would have started treatment that day if the mobile medical unit had not been present. Satisfaction scale and multiple-choice question responses were assessed using descriptive statistics. Wilcoxon signed-rank test was used to compare median satisfaction levels between receiving buprenorphine from the mobile medical unit versus filling a buprenorphine prescription at a community pharmacy. Open-ended questions were analyzed qualitatively using inductive thematic analysis.

**Results:**

106 unique patients were dispensed buprenorphine from the mobile unit during the study period. Of these patients, 54 (51%) completed the survey. Respondents reported high satisfaction with the buprenorphine dispensing process as a part of a mobile medical unit. Of those who had previously filled buprenorphine at a pharmacy, 83% reported at least one barrier, with delays in prescription dispensing from a community pharmacy, lack of transportation to/from the pharmacy, and opioid withdrawal symptoms being the most common barriers. 87% reported they would not have started buprenorphine that same day if the mobile medical unit had not been present. Nearly half of survey participants reported having taken buprenorphine that was not prescribed to them. Qualitative analysis of open-ended survey responses noted the importance of convenient accessibility, comprehensive care, and a non-judgmental environment.

**Conclusions:**

Mobile medical units that dispense buprenorphine are an innovative model to reach patients with OUD who have significant treatment access barriers. This study found that patients who experienced barriers to accessing buprenorphine from a pharmacy were highly satisfied with the mobile medical unit’s buprenorphine dispensing process. Programs seeking to develop mobile buprenorphine dispensing programs should consider patient priorities of accessibility, comprehensive care, and welcoming, non-judgmental environments.

**Supplementary Information:**

The online version contains supplementary material available at 10.1186/s13722-024-00484-4.

## Background

In the 12-month period ending in April 2023, there were over 106,000 US overdose deaths [1]. The rapid rise in overdose fatalities has been linked to the presence of high potency synthetic opioids, such as fentanyl, in the drug supply [2]. Medications for opioid use disorder (MOUD), particularly buprenorphine and methadone, are effective at reducing overdose events and symptoms of opioid use disorder (OUD) [3, 4]; however, less than 20% of people with OUD are engaged in treatment [5]. Disparities in access exist as well, with decreased access to MOUD for Black individuals with OUD compared to white individuals despite the disproportionate impact of opioid overdose deaths on Black individuals and communities [6, 7].

Increasing MOUD treatment access and patient engagement is critical to addressing the drug overdose crisis. A 2022 overview of systematic reviews found structural issues were the most cited barriers to substance use disorder (SUD) treatment, highlighting treatment provider service challenges, legal barriers, policy constraints, financial concerns, insufficient time, and inaccessibility of treatment programs [8]. People who use drugs (PWUD) may also experience housing insecurity, lack of transportation, and lack of health insurance coverage, thereby impacting their ability to access and remain engaged in treatment [9, 10]. In addition, mistrust in the health care system and treatment providers is common due to the variety of negative effects of stigma imposed on patients [11].

To address many of these barriers to care, low-threshold MOUD programs have emerged across the country, including programs integrated into harm reduction and street outreach programs [12–16]. In July 2021, the DEA updated regulations via 86 FR 33,861 to allow opioid treatment programs (OTPs), commonly known as methadone clinics, to dispense MOUD from mobile units as an extension of their OTP license [17]. The literature on MOUD mobile medical units is limited; however, initial studies suggest that mobile medical units providing MOUD can increase access to treatment, particularly for those facing structural barriers to care [18–22]. A recent implementation study of a mobile methadone program in San Francisco found that both staff and patients were satisfied with van-based methadone delivery and that the model streamlined visits, reduced wait times, and improved patient treatment experiences [23]. In Massachusetts, the Community Care in Reach program deployed mobile medical units to neighborhoods with high overdose rates with a goal of providing non-judgmental support, access to low-threshold MOUD, and harm reduction. Qualitative interviews with participants engaged by this program found the model to be highly acceptable by people who often experience stigma in traditional healthcare settings, as well as an emphasis on convenience and relationship-building. [16] Additionally, a recent qualitative study which interviewed program staff from five mobile MOUD programs in two different states (Ohio and Massachusetts) highlighted that mobile MOUD programs can improve immediate access to MOUD, advance equity, and decrease opioid overdose deaths. [24] Mobile MOUD programs and street-based outreach are particularly important strategies to engage people experiencing homelessness. This is critical, given that drug overdose is the leading cause of death in this population [25] and yet people experiencing homelessness are less likely to receive MOUD. [26]

The buprenorphine and methadone dispensation directly to patients from a mobile medical unit is novel and may address barriers that patients face when filling prescriptions for buprenorphine at a pharmacy. A 2021 audit of pharmacies in US counties with high overdose mortality rates found that nearly 20% of pharmacies indicated that they would not dispense buprenorphine [27]. A 2020 survey of patients who filled buprenorphine via a clinic-based pharmacy expressed that transportation challenges, time spent obtaining medication, feelings of stigma or shame, and lack of stock of medication were all barriers faced at community pharmacies [28]. In addition, one study found that patients who were given their prescribed buprenorphine at their office visit had over 50% higher retention after 6 months than those who were sent to a community pharmacy [29]. This paper seeks to add to the literature by exploring patient satisfaction with buprenorphine dispensing from a mobile medical unit, barriers experienced when filling buprenorphine prescriptions at pharmacies, and client-reported previous means of obtaining buprenorphine.

## Methods

### Community and program setting

In 2021, the Community Outreach Intervention Projects (COIP), a longstanding harm reduction program run by the University of Illinois Chicago (UIC) School of Public Health, collaborated with the UIC Colleges of Medicine and Pharmacy to develop and implement a mobile medical unit using a renovated recreational vehicle (RV) to provide low-threshold MOUD as well as primary care, wound care, and vaccination services [19]. The COIP mobile medical unit is staffed by an interprofessional team including harm reduction team members (including peers with lived experience), an outreach team member, a recovery support specialist, a clinical pharmacist, and a physician with addiction experience. Each team member supports all work in the field, but typically outreach workers will engage and register clients, the physician will assess patients, and the clinical pharmacist will engage with the physician and patient regarding medication selection and management as well as dispensing buprenorphine, where applicable.

The COIP mobile medical unit was deployed in areas with the highest opioid overdose rates in Chicago, in tandem with harm reduction services already being provided by COIP, regardless of client insurance or identification status. In addition, the clinic was furnished to provide anonymous HIV and hepatitis C testing in partnership with the Chicago Department of Public Health with pathways to link clients who use harm reduction services to specialized care where appropriate. Community partnerships were leveraged to provide access to social and mental health services to supplement clinical care.

Following the initial implementation of the mobile medical unit, clinical staff developed a partnership with a Chicago-based OTP to serve as a mobile OTP, allowing for the dispensing of buprenorphine dose packs directly from the mobile medical unit beginning in July 2022. Buprenorphine packs were dispensed as pre-made 2-day, 3-day, or 7-day supplies of buprenorphine/naloxone 8 − 2 mg sublingual films with directions to take twice daily with an additional film as needed, for up to three films sublingually daily (24 mg buprenorphine total). Buprenorphine dose packs were packaged by the clinical pharmacist in advance and stored at the OTP. On service days, the clinical pharmacist picks up prepackaged dose packs and returns unused dose packs to the OTP at the end of the shift.

### Study design

To assess client perceptions and satisfaction with receiving buprenorphine from the mobile medical unit, an anonymous 7-item survey was developed utilizing 5-point satisfaction scales (1 being “not satisfied at all” and 5 being “extremely satisfied”), a free response item related to reasons for their satisfaction level, and multiple-choice items related to previous methods of buprenorphine access and barriers to receiving buprenorphine from a community pharmacy. Patients who presented to the mobile medical unit and were dispensed buprenorphine between May 24, 2023, and August 25, 2023 were invited to take part in the uncompensated survey and verbally consented in compliance with UIC Institutional Review Board approved procedures. Only one survey response was collected from each unique participant. Patients who presented for non-OUD medical reasons (e.g. wound care, hypertension management, etc.) and those who presented for MOUD but were prescribed rather than dispensed buprenorphine were excluded from this study.

### Data collection

Following consent, a research team member read each survey question to the participant and recorded responses on paper, which typically took 5 to 10 min. Surveys were conducted privately on-site at the location of the mobile medical unit during clinic hours immediately after buprenorphine provision. Later, survey responses were transcribed into REDCap electronic data capture tools hosted at UIC for storage. Demographic data was not collected as part of the survey to preserve anonymity. Population cohort demographic data was collected via the electronic health record for all patients seen during this time for OUD treatment as a part of continuous quality measures of clinical service, regardless of whether they were dispensed buprenorphine from the mobile medical unit that day.

### Data analysis

Quantitative survey data were analyzed utilizing IBM SPSS Statistics v26 (Armonk, NY). The primary outcome was the difference in client satisfaction score of receiving buprenorphine from the mobile medical unit compared to a community pharmacy. Wilcoxon signed-rank test was used to compare median satisfaction levels between receiving buprenorphine from the mobile medical unit versus filling a buprenorphine prescription at a community pharmacy. Secondary outcomes included history of receiving buprenorphine from a community pharmacy, use of buprenorphine products without a prescription, likelihood of accessing buprenorphine products in the absence of the mobile medical unit, and barriers to receiving buprenorphine products from a community pharmacy. Descriptive statistics were used to assess secondary outcomes.

Inductive thematic analysis was conducted on qualitative survey data by three separate investigators to identify themes that reached thematic saturation. Following individual analysis, investigators met to reconcile identified themes and reach consensus.

## Results

### Mobile medical unit patient demographics and substance use history

During the three-month study period, 106 unique patients visited the mobile medical unit for OUD treatment and were dispensed buprenorphine. Of these, 54 (50.9%) completed the survey. The average client was 45 years old (SD = 11.1), Black (59.4%), male (70.8%), and insured (88.7%, 79.2% by Medicaid). The primary route of opioid use was via insufflation, and nearly 90% of patients have documented use of at least one other substance in addition to opioids, either by self-report or urine drug screen results. The mean duration of opioid use was over 18 years. See Table 1 for a full list of client demographics and substance use history.

**Table 1:** Population-based demographic information for patients accessing the mobile medical unit during the study period (*n* = 106)

Characteristic	*n* (%)
**Age – Years, mean (SD)**	**45.4 (11.1)**
**Gender**	
Male	75 (70.8)
Female	30 (28.3)
Non-binary	1 (0.9)
**Race/Ethnicity**	
Black or African American	63 (59.4)
White	30 (28.3)
Hispanic/Latinx	11 (10.4)
Other/unspecified	9 (8.5)
Unknown/uncollected	1 (0.9)
**Insurance Status**	
Insured	94 (88.7)
Insured by Medicaid	84 (79.2)
Uninsured	12 (11.3)
**Route of Substance Administration (** ***n*** ** = 94)**	
Insufflation only	67 (71.3)
Multiple routes	14 (14.9)
Injection only	11 (11.7)
Oral only	2 (2.1)
**Documented Polysubstance Use (** ***n*** ** = 88)**	
Polysubstance	79 (89.8)
Non-polysubstance	9 (10.2)
**Substance Use History**	
Bags used per day, mean (SD) - (*n* = 83)	4.7 (3.4)
Years of use, mean (SD) - (*n* = 78)	18.4 (12.2)

### Satisfaction of service

**Fig. 1:**
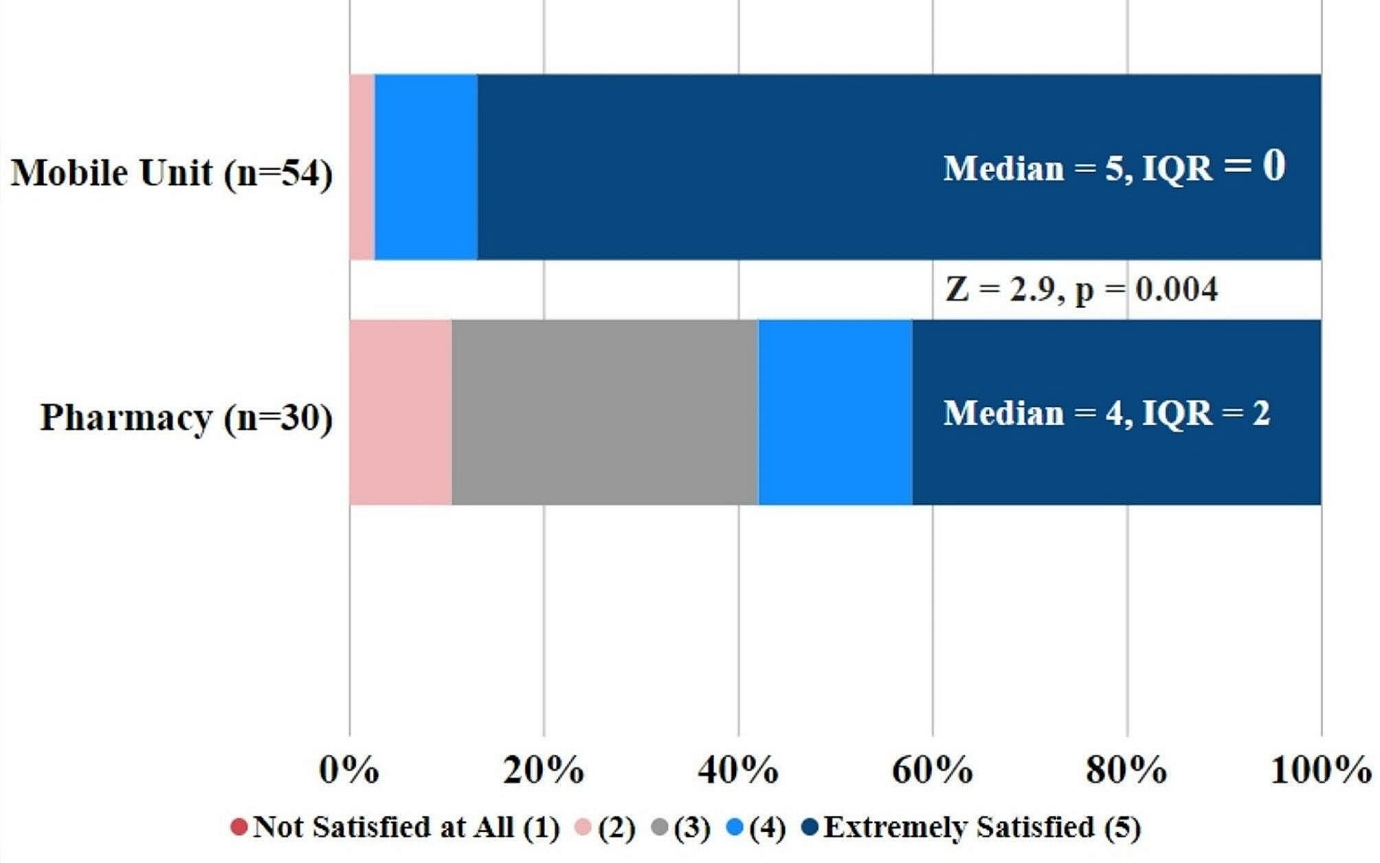
Client satisfaction with accessing buprenorphine from mobile medical unit versus a community pharmacy

Over half of respondents (*n* = 30) had previously received buprenorphine from a pharmacy, and 24 (44.4%) had previously accessed buprenorphine without a prescription. Respondents (*n* = 54) reported a median satisfaction of 5 (IQR = 0) with receiving buprenorphine from the mobile medical unit. Those who had previously received buprenorphine from a community pharmacy (*n* = 30) reported a median satisfaction of 4 (IQR = 2) for receiving buprenorphine from a community pharmacy (Z=-2.9, *p* = 0.004) (Fig. 1). Of those who previously received buprenorphine from a community pharmacy, 25 (83.3%) reported at least one barrier. Common barriers included delays in prescription dispensing (51.5%), lack of transportation to the pharmacy (54.5%), and experiencing opioid withdrawal symptoms (48.5%). See Table 2 for a full list of barriers endorsed by survey respondents. There were only 7 (13.0%) respondents who reported they would have accessed buprenorphine that day had the mobile medical unit not been present.

**Table 2:** Barriers to obtaining buprenorphine from a community pharmacy

Barrier	Number of Respondents (*n*)
Time delay in filling prescription	16
Lack of transportation to community pharmacy	15
Experiencing opioid withdrawal and unable to go to pharmacy	14
Lack of identification to pick-up prescription	10
No buprenorphine in stock at the community pharmacy	9
Stigma experienced by the patient	7
No pharmacy nearby	4
Pharmacist refused to fill prescription	1
Other (lack of money, pharmacy hours)	2

### Factors impacting satisfaction of buprenorphine dispensing

Thematic analysis revealed three major themes that drove level of satisfaction for buprenorphine dispensing: (1) accessibility; (2) comprehensive care; and (3) a non-judgmental setting.

### Theme 1: Accessibility: convenience is key for satisfaction

Many participants commented on the accessibility of the mobile unit location being a key component for their satisfaction with the buprenorphine dispensing process, as the mobile unit travels to the intersections with the highest overdose rates in the city. Multiple survey participants cited convenience as a major factor contributing to their satisfaction, as this area is challenged by community pharmacy deserts [30].

*Participant A: Convenient*,* keep up the good work*,* coming to ground zero where drug deals are happening is great. A lot of people would not want to travel to a clinic to get some*,* they’d say it’s too far and inconvenient to get to. [The team is] friendly*,* straight to the point*,* quick*,* answered questions*,* professional. I unfortunately come to this area a lot and it’s really convenient to have you here for us.*

Participants also mentioned factors such as not requiring insurance to be seen, a “walk in” model rather than appointment times, and efficient but high-quality service. They expressed a high priority for reduced wait times and readily accessible care, particularly in the neighborhoods where they live.

*Participant B: Super convenient*,* don’t have to wait. Being able to walk in is the best part.*

*Participant C: You’re awesome and I’ve been wanting to get on buprenorphine after being hospitalized recently*,* but I didn’t know where to get it*,* being uninsured*,* so this was truly a life-saver.*

*Participant D: Convenient. You don’t have to be too sick to come. You take [the mobile unit takes] everyone.*

### Theme 2: Comprehensive care: one stop shop

Patients seeking care at the mobile medical unit expressed a preference for the comprehensive approach to their care, including addressing needs beyond buprenorphine. Survey respondents commented on the benefit of receiving additional resources, such as food and clothing, during their visit to the mobile unit.

*Participant E: I appreciate everything you’re doing and the food*,* clothes*,* and resources.*

*Participant F: Good customer service; the doctor is always nice. We have good food – real food.*

Survey respondents also commented that the patient-centered, informative model was a driver for their high satisfaction, highlighting their appreciation for strong communication between the team and patients and ability to meet client needs.

*Participant G: Continue the great communication between patients and staff.*

*Participant H: They make sure they give me exactly what I want and need.*

### Theme 3: Non-judgmental approaches: meeting people where they are

Multiple survey respondents commented on the overall atmosphere of the mobile medical unit, particularly how the outreach and clinical team made them feel comfortable. The genuine concern from the staff for patients, support in recovery as a journey, and welcoming and positive attitude was a component of their high satisfaction with the service, denoting a preference for a non-judgmental space.

*Participant I: Introduction was made*,* it was nice. Everyone was polite and respectful.*

*Participant J: Helped [me] feel better and more comfortable; [they were] concerned when there is a slip to help*,* but not judge.*

*Participant K: It’s awesome y’all come out because some people like me are afraid to come to the clinic where I’m worried I’ll run into people I know*,* and I don’t have transportation*,* so it’s way more affordable and comforting.*

Additional themes that did not meet saturation included wait times and limitations related to clinical protocols, such as one client who expressed a preference for methadone, which was not available on the mobile unit. A summary of key findings with participant quotes is included in Table 3.

**Table 3:** Qualitative themes and exemplary quotes from client perspectives of accessing buprenorphine/naloxone from the mobile unit

Theme	Exemplary Quote
Accessibility: Convenience is key	*Convenient*,* keep up the good work*,* coming to ground zero where drug deals are happening is great. A lot of people would not want to travel to a clinic to get [treatment]*,* they’d say it’s too far and inconvenient to get to.* *I don’t have to wait in line*,* it’s pretty good.* *Friendly*,* straight to the point*,* quick*,* answered questions. I unfortunately come to this area a lot and it’s really convenient to have you here for us.* *Super convenient*,* don’t have to wait. Being able to walk in is the best part.* *Didn’t require insurance approval; staff is friendly and welcoming.* *You’re awesome and I’ve been wanting to get on buprenorphine after being hospitalized recently*,* but I didn’t know where to get it*,* being uninsured*,* so this was truly a life-saver.* *Convenient. You don’t have to be too sick to come. You take everyone.*
Comprehensive care: One stop shop	*I appreciate everything you’re doing and the food*,* clothes*,* and resources [you offer].* *Smooth*,* no bumps in the road*,* everyone is polite and met [my] needs.* *Good customer service*,* doctor is always nice. We have good food – real food.* *I prefer methadone but I know that’s not as accessible. For suboxone*,* this is about as good as it gets*,* though.* *Continue the great communication between patients and staff.* *They make sure they give me exactly what I want and need.*
Non-judgmental: Meeting people where they are	*It’s awesome y’all come out because some people like me are afraid to come to the clinic where I’m worried I’ll run into people I know*,* and I don’t have transportation*,* so it’s way more affordable and comforting.* *Everyone is nice and treats me nice*,* and I get good service.* *Introduction was made*,* it was nice. Everyone was polite and respectful.* *Helped [me] feel better and more comfortable. Concerned when there is a slip to help*,* but not judge.* *Staff is very genuine*,* stress-free*,* feel comfortable. I come from [other state] and their setting is great and it’s very positive here too.* *Other places made me jump through hoops.*

## Discussion

Overall, patients who received buprenorphine directly from the mobile medical unit reported high levels of satisfaction, with nearly 90% of survey respondents reporting feeling “extremely satisfied” with the buprenorphine dispensing process. Of those participants who had previously filled a buprenorphine prescription at a community pharmacy, they rated their experience on the mobile medical unit significantly higher. Survey participants listed several challenges to filling buprenorphine at community pharmacies in the past, highlighting delay times in filling medications, lack of transportation to/from the pharmacy, and significant opioid withdrawal symptoms limiting ability to go to a community pharmacy as barriers. In open-ended questions, many survey respondents cited convenience as a major positive factor in their satisfaction with the mobile medical unit, reflecting the need for elimination of these logistical barriers. Several participants endorsed stigma or feeling judged as past barriers experienced when accessing buprenorphine. These responses align with prior studies examining barriers to filling buprenorphine prescriptions and emphasize the benefit of putting medications directly into the hands of patients, meeting them where they are [8–10].

Recent policy changes to improve buprenorphine access, such as the removal of the X-waiver, increased telehealth access, and increased capabilities of OTPs to dispense MOUD in a mobile fashion, are positive [31]; however, this does not address barriers that arise for patients to initially access care or after a patient leaves the clinic to fill their prescription at a community pharmacy. Transportation support, increased coordination with community pharmacies to ensure adequate supply of buprenorphine, and streamlined procedures to fill medications more quickly may begin to address the barriers cited by participants. While policies have increased requirements for education of provider groups via the MATE Act of 2022 [32], pharmacists often lack the education regarding OUD during their training, and educational support would be useful to reduce stigma within community pharmacies and provide opportunities for other novel models of access to MOUD [33]. Initiatives to address pharmacy deserts as well as to address stigma faced at pharmacies are needed. Low-barrier programs providing buprenorphine, particularly those seeing patients in a street outreach model, should consider ways to address the barriers highlighted in this study, either by developing their own dispensing model or strengthening relationships with community pharmacies to address common challenges.

Nearly half of survey participants reported having taken buprenorphine that was not prescribed to them, either purchased or given to them by a friend. Use of non-prescribed buprenorphine is not a new phenomenon, and prior studies have found that most people who use non-prescribed buprenorphine do so primarily to self-manage opioid withdrawal symptoms or achieve abstinence from other opioids without engaging in a formal treatment program [34]. In fact, prior experience with non-prescribed buprenorphine has been associated with subsequent treatment entry and increased retention in treatment [35]. In addition, prior non-prescribed buprenorphine use may be associated with a decreased risk of complicated inductions or precipitated withdrawal, given the patient’s familiarity with this risk [36]. As non-prescribed buprenorphine use is common, it is important for treatment providers to establish a nonjudgmental environment in which patients can feel comfortable disclosing their use of non-prescribed buprenorphine, as well as consider how to incorporate non-prescribed buprenorphine use into medication counseling and treatment protocols. Survey respondents highlighted that they appreciated the non-judgmental, welcoming environment of the mobile medical unit which fosters this type of open communication. A key component of the COIP mobile medical unit is the integration of medical services into an existing harm reduction program that is led by outreach teams, with an emphasis on including outreach workers with lived experience. MOUD programs seeking to expand mobile operations should consider partnering with community-based harm reduction programs to help create a non-judgmental environment.

In addition, the COIP mobile medical unit notably saw a primarily Black patient population (nearly 60%). Opioid overdose deaths have disproportionality impacted Black communities, particularly in urban areas, which has worsened since the COVID-19 pandemic. [7] Despite this, studies have documented that Black individuals are significantly less likely to receive a buprenorphine prescription. [37] The ability of the COIP mobile MOUD program to effectively engage this population and reach a high level of participant satisfaction is important as we consider ways to address these inequities in access to MOUD.

Nearly 90% of survey participants reported that they would not have started treatment that day if the mobile MOUD program had not been present. This suggests that the mobile MOUD program successfully engages patients who otherwise would not have sought out care or would not have been able to access treatment due to health system or personal barriers. Given that over 80% of people with OUD are not engaged in treatment [5], this finding is critical to further explore. Access to buprenorphine within the community is harm reduction itself, as reflected in the decrease in overdoses generally in communities after buprenorphine became available [38]. Buprenorphine, being a partial opioid agonist, is protective in the setting of opioid use and an overdose episode [39], and there have even been case reports of sublingual buprenorphine being successfully used to reverse an opioid overdose [40]. In communities with high overdose rates such as where the COIP mobile medical unit provides care, dispensing low-threshold buprenorphine to people who are actively using illicit opioids is both a bridge into treatment as well as an extension of ongoing harm reduction efforts. Community harm reduction programs often serve as a critical linkage to low-barrier treatment with buprenorphine. Integrating buprenorphine initiation and dispensing into a harm reduction program takes the concept of meeting people where they are to the next level, allowing patients who otherwise face significant barriers to engage in treatment whenever they are interested.

### Limitations

Due to concerns about anonymity, the demographics of each survey respondent were not collected as part of the survey. Therefore, survey responses could not be linked to specific demographic factors or clinical outcomes. Without demographic data from the survey, we are not able to ensure that survey respondents appropriately represent the mobile unit client population. Additionally, surveys were offered and completed at the site of the mobile medical unit immediately after service delivery, which may have impacted survey responses. Although the surveys were administered by a research team member working separately from the clinical team, participants may have altered their responses due to the proximity of the clinical team, leading to social desirability bias. Non-response bias may have also impacted survey results, as an unsatisfied patient may be less likely to stay to complete the survey.

## Conclusions

Mobile medical units with the ability to dispense buprenorphine are an innovative model to reach patients who otherwise are not accessing care. Patients of a mobile buprenorphine program were very satisfied with the process of receiving buprenorphine from the mobile unit and further reported numerous barriers when filling prescriptions at community pharmacies. Programs seeking to develop mobile buprenorphine dispensing programs should consider client priorities of accessibility, comprehensive care, and welcoming, non-judgmental environments.

### Electronic supplementary material

Below is the link to the electronic supplementary material.

Supplementary Material 1

Supplementary Material 2

## Data Availability

The datasets used during the current study are available from the corresponding author on reasonable request.

## Abbreviations

OUD: Opioid Use Disorder
MOUD: Medications for Opioid Use Disorder
SUD: Substance Use Disorder
PWUD: People Who Use Drugs
OTP: Opioid Treatment Program
COIP: Community Outreach Intervention Projects
UIC: University of Illinois Chicago
