# Supplementary material for: Patient experiences of buprenorphine dispensing from a mobile medical unit
Source: Addict Sci Clin Pract. 2024 Jul 18;19:53. doi: 10.1186/s13722-024-00484-4 (PMC11264859; doi:10.1186/s13722-024-00484-4)
Supplement: Supplementary file 1 — Supplementary Material 1 [file 13722_2024_484_MOESM1_ESM.pdf]

Buprenorphine Dispensing Satisfaction Survey

1. How satisfied are you with receiving buprenorphine on the van today on a scale of 1 to 5, with 1 being not satisfied at all and 5 being extremely satisfied?
   Circle: 1 2 3 4 5

2. Why do you feel this way (either satisfied or not satisfied)?

3. Do you think you would have started buprenorphine today if the van had not been here?
   a. yes
   b. no

4. Have you taken buprenorphine that was prescribed to you at a pharmacy in the last year?
   a. yes
   b. no

   How satisfied have you been in the past with picking up a buprenorphine prescription from the pharmacy on a scale of 1 to 5, with 1 being not satisfied at all and 5 being extremely satisfied?
   Circle: 1 2 3 4 5

5. Have you ever taken buprenorphine that was not prescribed to you (such as from a friend or dealer)?
   a. yes
   b. no

6. Have any of the following ever been barriers you have faced to filling a prescription for buprenorphine at a pharmacy in the past?
   1. lack of transportation
   2. lack of ID
   3. banned from a particular pharmacy
   4. no pharmacy nearby
   5. pharmacist refused to fill prescription
   6. stigma/feeling judged
   7. feeling too sick/too much withdrawal to get to the pharmacy
   8. pharmacy not having buprenorphine in stock
   9. delay time in filling prescription/waiting at pharmacy
   10. other reasons:___________________________________

7. Any other feedback that you’d like to share?
