# Supplementary material for: Patient experiences of buprenorphine dispensing from a mobile medical unit
Source: Addict Sci Clin Pract. 2024 Jul 18;19:53. doi: 10.1186/s13722-024-00484-4 (PMC11264859; doi:10.1186/s13722-024-00484-4)
Supplement: Supplementary file 2 — Supplementary Material 2 [file 13722_2024_484_MOESM2_ESM.docx]

Dr. Casy Calver

*Addiction Science & Clinical Practice*

June 18, 2024

To Dr. Casy Calver:

We are submitting this second revision of our original research article titled “Patient Experiences of Buprenorphine Dispensing from a Mobile Medical Unit” for consideration by *Addiction Science & Clinical Practice*. Thank you for the feedback provided on our revised manuscript; the suggestions to clarify our methods have added clarity and strengthened the manuscript.

In this revised version, we have added more detail to the abstract methods section, as well as clarified our analysis in the methods section. We have submitted a point-by-point response to outline the recommended changes. There are no conflicts of interest for any research team members. We have made no changes to the authors or author order for the manuscript.

Please address all correspondence regarding this manuscript to me at messmer2@uic.edu.

Thank you for considering our manuscript for publication. Please do not hesitate to contact me with any questions or clarifications.

Sincerely,

Sarah Messmer, MD

Assistant Professor of Clinical Medicine and Pediatrics

University of Illinois at Chicago

Westside Research Office Building, Rm 256

1747 W Roosevelt Rd

Chicago, IL 60608

Phone: 815-276-7579

Email: messmer2@uic.edu
